# Reliability and Construct Validity of the SENS Motion® Activity Measurement System as a Tool to Detect Sedentary Behaviour in Patients with Knee Osteoarthritis

**DOI:** 10.1155/2018/6596278

**Published:** 2018-03-01

**Authors:** Cecilie Bartholdy, Henrik Gudbergsen, Henning Bliddal, Morten Kjærgaard, Kasper Lundberg Lykkegaard, Marius Henriksen

**Affiliations:** ^1^The Parker Institute, Copenhagen University Hospital, Bispebjerg and Frederiksberg, Copenhagen, Denmark; ^2^Department of Physical and Occupational Therapy, Copenhagen University Hospital, Bispebjerg and Frederiksberg, Copenhagen, Denmark; ^3^SENS Innovation Aps, Copenhagen, Denmark

## Abstract

Physical inactivity is important to address, and an objective way of measuring inactivity is by accelerometry. The objective of this study was to determine the reliability and construct validity of the SENS motion system to record physical activity and inactivity in patients with knee osteoarthritis. Participants with an age > 40 years and an average weekly pain above 0 on a numeric rating scale (0 = no pain, 10 = worst pain) were included. Participants had a total of two study visits and at each visit participants completed a standardized activity. Data from 24 participants were analysed. A mean agreement of 99% (SD 3%) for sedentary behaviour and a mean agreement of 97% (SD 9%) for active behaviour were found. The agreement for “walking” was 28% (SD 18%). Mean agreement between recordings on the two visits was 96% (SD 8%) for sedentary behaviour and 99% (SD 1%) for active behaviour. The SENS motion activity measurement system can be regarded as a reliable and valid device for measuring sedentary behaviour in patients with knee OA, whereas detection of walking is not reliable and would require further work.

## 1. Introduction

Physical inactivity is related to poor health status and associated with higher all-cause mortality rates [1]. National and international guidelines [2, 3] highlight the importance of regular moderate to vigorous physical activity and minimized sedentary behaviour to achieve a general good health [4–6]. Reliable, easy, inexpensive, and objective means of measuring physical activity over time in different populations is therefore of great interest. Information about individuals physical activity patterns will help us understand how sedentary behaviour influences health and may be used to support preventive measures against inactivity related to chronic illness.

A widely accepted way of measuring physical activity is by accelerometry. Accelerometers measure type, frequency, duration, and intensity of physical activity. Accelerometry is considered superior to other methods such as pedometers, heart rate monitors, or questionnaires regarding validity and specificity of the physical activity measurements [7].

Recent advances in technology have reduced cost and size of accelerometers [8]. Miniaturized accelerometers in combination with smartphone technology, better data quality, longer battery lifetimes, and better data storage opportunities have led to improved user-friendliness and expanded possibilities of data collection and usage. Altogether, the advantages of using accelerometers to record type and level of physical activity have been enhanced, ultimately increasing the feasibility of activity measurement systems in both research and clinical settings. 

The SENS motion activity measurement system (SENS motion system) is a recent example of a physical activity measurement system that exploits the above advantages. It is a waterproof miniature accelerometer placed within a small band-aid to be worn discretely on the thigh. The sensor continuously records the orientation and acceleration of the thigh and synchronizes with a smartphone app that uploads the recorded data to a secure web server for storage and analysis. A predefined algorithm integrates the sensor's orientation with respect to gravity and the recorded acceleration and categorizes the recordings into predefined types of physical activities: lying down, sitting, standing, walking, and other activities. While feasibility of such technology seems obvious, the validity and reliability of the SENS motion system have not yet been determined.

A common population known to be less physically active than their healthy age-matched controls is people with knee osteoarthritis (OA) [9], where knee pain and other symptoms may prevent patients in engaging in physical activity. This population is particularly vulnerable to inactivity related comorbidities, as there is an overrepresentation of overweight and obesity among people impacted by knee OA. By consequence, it would be highly relevant to assess validity and reliability of the SENS motion system in this population, because a feasible physical activity measurement system could be used to facilitate physical activity with potential beneficial effects on general health.

The purpose of this study was to determine the construct validity and reliability of the daily activity measurement system and its accompanying algorithm for physical activity estimations (the SENS motion system) by comparing data recorded by the sensor with controlled observations and self-reported activities in individuals with knee OA.

## 2. Methods

### 2.1. Participants

Participants were included via an in-house patient register at the OA outpatient clinic at the Parker Institute, Frederiksberg Hospital, Copenhagen, Denmark. Each participant was contacted by telephone or email and invited to participate and the details of the project were explained. All interested individuals were screened for eligibility and gave informed consent before inclusion in the study. The eligibility criteria were as follows: age > 40 years and a diagnosis of knee OA by a rheumatologist according to the diagnostic criteria as described by the American College of Rheumatology [10]. Exclusion criteria were inability to safely walk on a treadmill, transfer from lying to standing position without assistance, or walk independently without a walking aid.

Participant data was handled according to Danish law regarding patient sensitive data storage and the SENS motion system was approved by the Danish Data Protection Agency (j.no. 2012-58-0004). According to Danish law on Health Research Ethics Committee this project did not require approval by the committee, which was confirmed by the Regional Health Research Ethics Committee (case number 15018244) and participants were not required to sign an informed consent form.

### 2.2. Study Design

An observational study design was used to assess the validity and reliability of the SENS motion system by measuring the participants' activity patterns over a timespan of approximately 24 hours. During study participation, the participants had two study visits at the facility (Parker Institute). At visit one, general demographics were recorded and the sensor was mounted and activated (see below) before commencing a set of standardized activities in a controlled environment following a standardized protocol (described below). After completing the standardized protocol, participants were sent home and instructed to fill in a physical activity diary for the next 24 hours (semistandardized protocol). The standardized and semistandardized protocols were used to assess validity (precision) of the SENS motion system.

At visit two, the standardized activities were repeated with the sensor placed on the contralateral thigh, in order to assess reliability of contralateral measurements. The replacement of the sensor was done unsupervised by the participants (i.e., without additional instructions) to simulate replacement in “real world” conditions.

### 2.3. The SENS Motion System

The SENS motion system consists of a waterproof sensor (50 × 21 × 5 mm, weight 8 g), with a triaxial accelerometer, sampling acceleration at 12.5 Hz, with a range of ±4*G*, connected wirelessly (Bluetooth technology) to a dedicated smartphone application (both android and iOS available). The sensor is embedded within a band-aid (Medipore™, 3M, Soft Cloth Surgical Tape on Liner) and attached to the skin on the lateral aspect of the thigh, approximately 10 centimetres from the lateral epicondyle of the knee. The sensor has a battery lifespan of approximately 20 weeks. The hardware version used was SENS motion plus version 1.3.0 with sensor firmware version 1.0.2. The sensor has a built-in memory capacity to store data for 14 days if the sensor is out of reach of a smartphone. The dedicated software application is installed on the user's smartphone or tablet and using Bluetooth technology the sensor connects to the smartphone (or smart device) when in use and within necessary range. The raw data recorded by the sensor is transmitted to the smartphone app every 10 seconds, unless the sensor is out of reach, in this case data is stored on the sensor until connected. When raw data has been transmitted to the app, it is automatically uploaded to a secure web server, whenever the smartphone connects to a Wi-Fi Internet access point.

### 2.4. Position and Activity Detection

A predefined algorithm for detection of different categories of activity was used to analyse data. The algorithm integrates the orientation of the sensor and the recorded acceleration.

Orientation is estimated by use of a gravity vector obtained from the acceleration signal, defined as the angle of the average acceleration signal over a 10-second interval. The orientation estimates range from 0 radians to *π*/2 (1.57) radians, where *π*/2 radians define a vertical position, and 0 refers to horizontal. To discern between activities done during upright position, a predefined threshold of 0.75 radians was used to discriminate between predominantly vertical positions of the thigh (e.g., standing, walking, and stair climbing) and predominantly horizontal positions of the thigh (e.g., sitting and lying down).

The type of activity was assessed by determining the intensity of the motion performed. This intensity was calculated based on the sum of the squared acceleration of all three axes (*G*^2^) compensated for the static gravity component, by averaging the maximum peak to peak amplitude in 2-second windows over a period of 10 second interval. The intensity of the acceleration was divided into three categories, below 0.3*G*^2^, between 0.3*G*^2^ and 1.3*G*^2^, and above 1.3*G*^2^. These cut-off values was used to discriminate between the intensity of the thigh movement, where anything over 1.3*G*^2^ was considered activities with higher intensity than walking and anything below 0.3*G*^2^ was considered too low to represent movement. This algorithm was developed, based on early experiences and algorithm development with the SENS motion system on healthy people.

The algorithm combines the orientation and acceleration to generate the following four categories of activity: “sedentary” (lying down and sitting), “standing,” “walking,” and “other activities.” Table 1 shows the cross-tabulation of thresholds used to categorize physical activity. We further dichotomized the build-in categories in to “sedentary” (lying down and sitting) and “active” (standing, walking, and other activities all together) behaviours.

### 2.5. Standardized Physical Activity Protocol

During the clinical visits, the participants performed a series of activities observed and supervised by a researcher (CB) who observed and recorded the exact times of each of the different prespecified activities. Each participant completed the same activities in the same order: lying down (2 min), sitting (2 min), standing (2 min), walking on a treadmill (at least 2 min, at a self-selected pace), walking outside (5 min), walking up and down stairs (20 sec), and biking on a stationary ergometer bike (2 min).

The different activities were chosen for their common daily use. Similarly the SENS motion system algorithm categorized the activities as “sedentary” (lying down and sitting), “walking” (including walking on a treadmill and walking outside), “standing,” and “other activities” (including stair walking and biking).

The activity protocol was repeated at visit two, with the sensor replaced on the contralateral thigh.

### 2.6. Semistandardized Protocol

Participants were requested to fill in a 24-hour activity diary (see additional file 1). The participants were instructed to record type of activity and date and time of day as frequently as possible. Both oral and written instructions were given to the participants before they were sent home. The reported activities were then categorized by CB into the before mentioned categories: “sedentary” (lying down and sitting), “walking” (including walking on a treadmill and walking outside), “standing,” and “other activities” (e.g., stair walking and biking) to make comparison between data from the SENS motion system and the diary possible.

### 2.7. Feasibility Questionnaire

At the end of study visit two, the participants' were asked to fill out a feasibility questionnaire in order to evaluate the user-friendliness of the SENS motion system. The questionnaire consisted of nine questions, each rated on a Likert rating scale with 5 levels of agreement, from “strongly agree” to “strongly disagree.” The questionnaire is displayed in Table 2.

### 2.8. Statistics

All analyses were based on participants who completed all evaluations, that is, observations at visit one and two, diary recordings, and the feasibility questionnaire. All of the statistical analyses were carried out in Microsoft Excel 2010.

Physical activity data was analysed in two different ways; first we analysed the dichotomized behaviours (“sedentary” and “active”). Secondly we analysed data according to the four predefined categories: sedentary, standing, walking, and other activities. The different activities were categorized in bouts of 10 seconds. The agreement between sensor-based categorizations, observations (standardized protocol), and diaries (semistandardized protocol) was assessed by percentage of agreement.

To assess reliability, the sensor data obtained from the two study visits was compared. The different activities were summarized in bouts of 10 seconds and agreement between the two study visits was assessed by percentage of agreement.

The questionnaire scorings were analysed as follows: scores (answers) from each question was averaged across all participants. An average score below 3 in questions (1)–(3) and a score above 3 in questions (4)–(9) would suggest good feasibility and user-friendliness of the sensor for this patient group.

## 3. Results

A total of 27 patients with knee OA agreed to participate in this study, of these 23 had full data sets. One participant had missing data during the standardized stair walking at visit two but was still included in the overall assessment of validity and reliability, resulting in 24 participants included in the final analysis. The remaining 3 participants had missing data for longer periods (several hours) due to technical issues with the sensor during the transmission from the sensor to the app or from the app to the web server. Patient demographics of the 24 participants are described in Table 3.

### 3.1. Construct Validity

#### 3.1.1. Standardized Physical Activity Protocol

A mean agreement of 97% (SD 7%) was observed between the observations and SENS motion system when using the dichotomized categories “sedentary” and “active” behaviours. When using the 4 predefined categories, a mean agreement of 53% (SD 11%) between the observations and the SENS motion system was observed. The lowest percentage of agreement was for “walking” with a mean of 28% (SD 18%) and the highest percentage of agreement was for “sedentary” with a mean of 99% (SD 3%).  Table 4 presents the agreement scores between the observations and the SENS motion system for each participant, at each study visit, in each category.

#### 3.1.2. Semistandardized Protocol

A mean agreement of 92% (SD 5%) was observed between the diaries and the SENS motion system when using the dichotomized categories “sedentary” and “active” behaviours. When grouping the data into the 4 predefined categories, a mean agreement of 88% (SD 5%) was observed. The lowest percent agreement was 42% (SD 36%) for the category “other activities” and the highest percent agreement was 94% (SD 5%) for the category “sedentary.” Table 5 presents the agreement scores between the semistandardized recordings and the SENS motion system for each participant and for each category.

### 3.2. Reliability

A mean agreement of 98% (SD 3%) was observed between the recordings obtained with the SENS motion system from the two study visits when using the dichotomized categories “sedentary” and “active” behaviours. When grouping the data into the 4 predefined categories, a mean agreement of 83% (SD 10%) was observed. The lowest percent agreement for the latter approach for categorization was 77% (SD 14%) for the category “walking” and the highest percent agreement was 96% (SD 8%) for the category “sedentary”; see Table 6 for percent agreement for each participant in each category.

### 3.3. Feasibility Questionnaire

Regarding feasibility of the SENS motion system the average score in question (1)–(3) was below 3 and above 3 in question (4)–(9) suggesting good feasibility and user-friendliness (Table 7).

## 4. Discussion

The aim of this study was to assess the construct validity and reliability of the SENS motion system in patients with knee OA.

The SENS motion system delivered valid recordings, both in the standardized and in semistandardized environments, when the algorithm was set to differentiate between “sedentary” and “active” behaviours. The agreement scores between the observations and the SENS motion system from both protocols were regarded as high indicating that the data obtained from the SENS motion system on sedentary behaviour would in fact reflect the actual level of sedentary behaviour. In terms of detecting body postures and activity, overall agreement between sedentary and active behaviour in the standardized environment appears to be as accurate as other accelerometer based monitors [11–13].

The SENS motion system's ability to distinguish between the 4 predefined categories (sedentary, standing, walking, and other activities) was not as consistent as the dichotomized differentiation between sedentary and active behaviours on the basis of observations in a controlled environment. Particularly, walking was misclassified as “other activities” suggesting that the upper limit of the acceleration pattern limits for walking may be too low for this population. This was not the case when comparing the sensor data with the diaries; here the average agreement for “other activities” was the lowest. This suggests that the algorithm's lower limit for categorizing “other activities” may be too high.

If the SENS motion system is used over a longer period of time the, data differentiating between the types of activity, for example, time spent on walking or other activities, should be interpreted with caution. It is possible that a systematic misclassification of behaviour with the existing algorithm will either under- or overestimate time spent on these specific activities.

However, studies have shown that the total time spent sitting plays an important role in the individual's general health [14, 15], not only intensity or type of activity. People with knee OA are generally less physically active than their healthy peers [16] suggesting that a decrease in sedentary time—irrespective of type of activity—is beneficial. In that perspective, this study shows that the SENS motion system is capable of distinguishing between sedentary and active behaviour with a consistently high accuracy making it a valuable device to monitor sedentary behaviour in the knee OA population. However, the detection of walking was poor. The detection of walking as a physical activity would be valuable, as walking disability is a risk factor of cardiovascular disease and death in patients with hip and knee OA [17, 18]. More advanced data interpretation methods should be further researched to enable a more reliable detection of walking.

When comparing data from the SENS motion system recorded on the two study visits (standardized protocol), the agreement was very high (almost identical) suggesting that repositioning of the sensor during an observation period is unlikely to affect the overall output. A minor disagreement between the two measurement days was observed for the category “walking” indicating that comparisons of two different recordings on the same subject will have a small variation in the estimation of walking time. However the overall agreement between the recordings from the two study visits suggests that the SENS motion system can be regarded as a reliable measurement system for measuring (in)activity and body position despite repositioning the device during a prolonged observation period. Repositioning of the sensor may be necessary as the sensor is attached by a band-aid, which may cause local skin irritations. The present study indicates that the sensor can be replaced by the participant, without affecting the precision of the recordings of physical (in)activity. Furthermore no association was found between BMI and the different agreement scores suggesting that data from the sensor is unlikely to be influenced by BMI.

The feasibility/user-friendliness questionnaire showed a generally positive attitude among the participants towards the SENS motion system, in terms of wearing the sensor and the participants' perceived influence on the participants' routines of daily life. This indicates that the sensor is of minimal inconvenience, which most likely is attributed to the sensor's size, water-resistance, and discrete placement. Altogether the results of this study point towards the SENS motion system as an attractive device to use in both research and clinical settings.

### 4.1. Study Limitations

A limitation in this study is the algorithm used to categorize the different activities. The algorithm was based on observations in healthy subjects during initial development of the sensor and algorithm. This may have influenced the accuracy of the SENS motion system data output when using the system on individuals with knee OA. Indeed, the algorithm misclassified walking in both the standardized and diary based phases of this study. Previous studies have suggested that one algorithm for all users is too unspecific [19]. Development of a specific algorithm for this patient group or personalized calibration may increase the accuracy of the SENS motion system's ability to categorize the different activities accurately. Another limitation of this study is the lack of observation in true daily life settings. The applicability of the SENS motion system in a true daily life setting remains to be determined. It is not unlikely that the results would be different if the observations were performed in other settings. However, we believe that the semistandardized protocol used in this study covers the most basic movement patterns involved in everyday activities.

## 5. Conclusion

The SENS motion system can be regarded as a reliable and valid device for measuring sedentary behaviour in patients with knee OA. The system's ability to distinguish between activity, standing, and sedentary behaviour is good and consistent. However, it is less consistent when differentiating between walking and other activities. The type of activity (walking and other activities) identified by the algorithm used in this study should be interpreted with caution. Further work should be done on the algorithm to increase its specificity regarding different activity patterns and different populations. 

## Figures and Tables

**Table 1:** Algorithm for identification of the different body positions or movements.

	Orientation < 0.75	Orientation ≥ 0.75
Acceleration < 0.3	Sedentary	Standing
0.3 < acceleration < 1.3		Walking
1.3 < acceleration	Other activities	

**Table 2:** Feasibility questionnaire: this was handed out to the participants at the end of study visit two and they were asked to respond by marking their answer to each question with an X.

Feasibility questions	Strongly agree	Agree	Undecided	Disagree	Strongly disagree
(1) The activity monitor is easy to remove					

(2) The activity monitor fits easily underneath clothing					

(3) I forgot I was wearing the monitor					

(4) I noticed wearing the monitor while doing my daily activities					

(5) The activity monitor limits me during my daily activities					

(6) The activity monitor limits me when I'm exercising					

(7) I have been more active because of the activity monitor					

(8) I have been less active because of the activity monitor					

(9) I would be ashamed if others would see I was wearing the activity monitor					

**Table 3:** Demographics.

Variables	Mean (SD)	Median (range)
Gender, number of women (%)	18 (75)	
Age, years	67.3 (6.5)	67 (56, 79)
Height, cm	169.9 (9.2)	168 (158, 192)
Weight, kg	82.3 (14.4)	81.5 (57, 116)
BMI, kg/m	28.5 (4.2)	28.08 (20.86, 37.32)
Average pain during the last week (NRS)	3.3 (1.9)	3.5 (1, 8)
Affected knee		
Left knee	5	
Right knee	9	
Both knees	10	

**Table 4:** Agreement between observations in a controlled environment, via the application of the standardized physical activity protocol at study visits one and two, and the SENS motion system as the percentage of agreement for each participant and for the entire cohort (median and range).

Number	Study visit	Sedentary	Standing	Walking	Other activities	Total	Active	Sedentary	Total
(1)	1	96%	100%	59%	67%	**68%**	99%	96%	**99%**
	2	100%	92%	17%	100%	**43%**	100%	100%	**100%**
(2)	1	100%	92%	39%	91%	**65%**	100%	100%	**100%**
	2	96%	92%	24%	90%	**53%**	100%	100%	**100%**
(3)	1	100%	92%	46%	100%	**65%**	99%	100%	**99%**
	2	100%	92%	44%	-	**62%**	98%	100%	**98%**
(4)	1	100%	100%	13%	64%	**39%**	100%	100%	**100%**
	2	96%	92%	42%	88%	**59%**	100%	96%	**99%**
(5)	1	100%	100%	11%	55%	**56%**	100%	100%	**100%**
	2	100%	100%	18%	22%	**53%**	90%	100%	**93%**
(6)	1	96%	100%	2%	100%	**33%**	87%	96%	**88%**
	2	100%	92%	8%	100%	**42%**	100%	100%	**99%**
(7)	1	100%	92%	40%	100%	**65%**	100%	100%	**100%**
	2	100%	92%	31%	89%	**59%**	100%	100%	**100%**
(8)	1	96%	100%	48%	100%	**64%**	50%	100%	**59%**
	2	100%	92%	22%	100%	**51%**	66%	100%	**74%**
(9)	1	100%	100%	7%	100%	**37%**	100%	100%	**100%**
	2	100%	100%	0%	100%	**41%**	100%	100%	**100%**
(10)	1	96%	75%	54%	22%	**61%**	94%	96%	**94%**
	2	100%	92%	38%	29%	**53%**	97%	100%	**98%**
(11)	1	100%	100%	6%	83%	**49%**	98%	100%	**99%**
	2	100%	100%	2%	100%	**49%**	98%	100%	**99%**
(12)	1	83%	100%	43%	100%	**59%**	100%	83%	**97%**
	2	100%	100%	40%	100%	**58%**	100%	100%	**100%**
(13)	1	100%	100%	33%	100%	**54%**	100%	100%	**100%**
	2	100%	100%	32%	100%	**52%**	100%	100%	**100%**
(14)	1	100%	100%	57%	100%	**70%**	100%	100%	**100%**
	2	100%	83%	23%	100%	**46%**	98%	100%	**99%**
(15)	1	100%	100%	60%	80%	**72%**	99%	100%	**99%**
	2	100%	92%	13%	63%	**38%**	96%	100%	**96%**
(16)	1	96%	92%	3%	100%	**33%**	100%	96%	**99%**
	2	100%	92%	36%	100%	**59%**	100%	100%	**99%**
(17)	1	96%	100%	36%	100%	**56%**	100%	96%	**99%**
	2	100%	100%	35%	100%	**56%**	100%	96%	**100%**
(18)	1	96%	100%	0%	100%	**33%**	100%	100%	**99%**
	2	100%	92%	6%	88%	**36%**	100%	100%	**100%**
(19)	1	100%	100%	1%	100%	**33%**	98%	100%	**100%**
	2	100%	83%	11%	88%	**38%**	100%	100%	**98%**
(20)	1	100%	92%	9%	100%	**34%**	100%	100%	**100%**
	2	100%	92%	41%	100%	**59%**	100%	96%	**100%**
(21)	1	96%	92%	55%	100%	**69%**	100%	96%	**99%**
	2	96%	100%	33%	100%	**55%**	100%	100%	**99%**
(22)	1	100%	100%	22%	100%	**55%**	100%	100%	**100%**
	2	100%	100%	31%	100%	**59%**	99%	100%	**100%**
(23)	1	100%	92%	39%	100%	**58%**	98%	100%	**99%**
	2	100%	83%	28%	100%	**50%**	98%	100%	**99%**
(24)	1	100%	92%	58%	78%	**69%**	99%	100%	**99%**
	2	100%	92%	50%	100%	**65%**	100%	99%	**99%**

*Median*		100%	92%	31%	100%	**55%**	100%	100%	**99%**
*Range*		83–100%	75–100%	0–60%	22–100%	**33–72%**	50–100%	83–100%	**59–100%**
*Mean*		99%	95%	28%	89%	**53%**	97%	99%	**97%**
*SD*		3%	6%	18%	21%	**11%**	9%	3%	**7%**

-: missing observation.

**Table 5:** Agreement between the semistandardized protocol, captured via diaries, and the SENS motion system as the percentage of agreement for each participant and for the entire cohort (median and range).

Number	Sedentary	Standing	Walking	Other activities	Total	Active	Sedentary	Total
(1)	95%	76%	85%	0%	**87%**	87%	95%	**87%**
(2)	97%	93%	93%	97%	**89%**	93%	97%	**91%**
(3)	98%	89%	82%	84%	**92%**	87%	97%	**94%**
(4)	85%	76%	27%	2%	**75%**	64%	85%	**81%**
(5)	88%	56%	90%	0%	**88%**	76%	89%	**90%**
(6)	97%	97%	92%	62%	**90%**	92%	97%	**94%**
(7)	100%	91%	52%	0%	**97%**	96%	100%	**99%**
(8)	91%	86%	93%	32%	**84%**	79%	91%	**88%**
(9)	90%	79%	76%	76%	**84%**	85%	90%	**93%**
(10)	79%	43%	60%	13%	**77%**	60%	79%	**83%**
(11)	96%	76%	91%	6%	**93%**	80%	96%	**95%**
(12)	95%	83%	100%	0%	**93%**	91%	96%	**94%**
(13)	93%	81%	79%	86%	**88%**	84%	93%	**91%**
(14)	88%	77%	52%	74%	**84%**	64%	88%	**86%**
(15)	97%	85%	95%	69%	**93%**	90%	97%	**95%**
(16)	97%	90%	80%	7%	**84%**	90%	97%	**92%**
(17)	97%	92%	50%	85%	**91%**	80%	97%	**93%**
(18)	98%	92%	86%	87%	**92%**	96%	98%	**96%**
(19)	97%	78%	55%	13%	**88%**	93%	97%	**96%**
(20)	96%	81%	84%	59%	**87%**	90%	96%	**90%**
(21)	92%	95%	62%	0%	**86%**	80%	92%	**88%**
(22)	99%	100%	70%	36%	**94%**	98%	99%	**97%**
(23)	97%	93%	94%	77%	**91%**	95%	97%	**95%**
(24)	94%	98%	92%	48%	**93%**	89%	94%	**96%**

*Median*	96%	85%	83%	42%	**89%**	88%	96%	**93%**
*Range*	79–100%	43–100%	27–100%	0–97%	**75–97%**	60–98%	79–100%	**81–99%**
*Mean*	94%	84%	77%	42%	**88%**	85%	94%	**92%**
*SD*	5%	13%	19%	36%	**5%**	11%	5%	**5%**

**Table 6:** Percentage of agreement between the SENS motion system data recorded at the two study visits while performing the standardized protocol with mean, median, standard deviation (SD), and range.

Number	Sedentary	Standing	Walking	Other activities	Total	Active	Sedentary	Total
(1)	96%	83%	45%	50%	**57%**	99%	96%	**99%**
(2)	96%	83%	73%	93%	**81%**	98%	96%	**97%**
(3)	100%	100%	75%	-	**83%**	99%	100%	**99%**
(4)	96%	92%	71%	75%	**78%**	99%	96%	**99%**
(5)	63%	100%	77%	67%	**75%**	96%	63%	**86%**
(6)	96%	92%	90%	100%	**92%**	100%	96%	**99%**
(7)	100%	83%	73%	89%	**81%**	99%	100%	**99%**
(8)	96%	92%	51%	100%	**68%**	100%	96%	**99%**
(9)	100%	100%	91%	100%	**95%**	100%	100%	**100%**
(10)	96%	83%	82%	14%	**81%**	95%	96%	**95%**
(11)	100%	100%	91%	83%	**94%**	98%	100%	**99%**
(12)	83%	100%	89%	100%	**90%**	100%	83%	**97%**
(13)	100%	100%	88%	100%	**92%**	100%	100%	**100%**
(14)	100%	83%	70%	100%	**78%**	98%	100%	**99%**
(15)	100%	92%	50%	80%	**64%**	97%	100%	**98%**
(16)	96%	100%	67%	100%	**78%**	100%	96%	**99%**
(17)	96%	100%	91%	100%	**93%**	100%	96%	**99%**
(18)	96%	92%	94%	88%	**94%**	100%	96%	**99%**
(19)	100%	83%	90%	88%	**91%**	98%	100%	**98%**
(20)	100%	83%	58%	100%	**70%**	100%	100%	**100%**
(21)	96%	100%	78%	100%	**84%**	100%	96%	**99%**
(22)	100%	100%	73%	100%	**84%**	100%	100%	**100%**
(23)	100%	92%	87%	100%	**90%**	99%	100%	**99%**
(24)	100%	100%	88%	88%	**91%**	99%	100%	**99%**

*Median*	98%	92%	77%	100%	**84%**	99%	98%	**99%**
*Range *	63–100%	83–100%	45–94%	14–100%	**57–95%**	95–100%	63–100%	**86–100%**
*Mean*	96%	93%	77%	88%	**83%**	99%	96%	**98%**
*SD*	8%	7%	14%	21%	**10%**	1%	8%	**3%**

-: missing value.

**Table 7:** The answers to the nine questions from the feasibility questionnaire are presented with mean, median, and range for each question.

(1) The activity monitor is easy to remove	Mean	1,1
	Median	1
	Range	1-2

(2) The activity monitor fits easily underneath clothing	Mean	1,0
	Median	1
	Range	1-2

(3) I forgot I was wearing the monitor	Mean	1,6
	Median	1
	Range	1–5

(4) I noticed wearing the monitor while doing my daily activities	Mean	4,4
	Median	5
	Range	1–5

(5) The activity monitor limits me during my daily activities	Mean	4,9
	Median	5
	Range	4-5

(6) The activity monitor limits me when I am exercising	Mean	4,7
	Median	5
	Range	3–5

(7) I have been more active because of the activity monitor	Mean	4,0
	Median	4
	Range	2–5

(8) I have been less active because of the activity monitor	Mean	4,3
	Median	4
	Range	3–5

(9) I would be ashamed if others would see I was wearing the activity monitor	Mean	4,8
	Median	5
	Range	3–5

## Supplementary Materials

Supplementary Materials Supplementary description: the 24-hour activity diary that participants were asked to fill out, as part of the semistandardized protocol. The participants were instructed to record type of activity, date, and time of day as frequently as possible during the 24-hour recording period.
