# Supplementary material for: Reliability and Construct Validity of the SENS Motion® Activity Measurement System as a Tool to Detect Sedentary Behaviour in Patients with Knee Osteoarthritis
Source: Arthritis. 2018 Mar 1;2018:6596278. doi: 10.1155/2018/6596278 (PMC5852870; doi:10.1155/2018/6596278)
Supplement: Supplementary Materials — Supplementary description: the 24-hour activity diary that participants were asked to fill out, as part of the semistandardized protocol. The participants were instructed to record type of activity, date, and time of day as frequently as possible during the 24-hour recording period. [file 6596278.f1.docx]

| Copenhagen University hospital, Bispebjerg and Frederiksberg |
| --- |
| 24 hour activity diary |
| The Parker Institute |
|  |
|  |
|  |

| During the next 24 hours you should fill out this diary with details about your activities. We would like to ask you to write down time of the day and for how long you spent on the different activities. An example could be: from 23.00 until 07.00, sleeping, from 08.45 until 09.30 eating breakfast. Please call on this number, 38 16 41 61 if you have any questions. |
| --- |

| **Time** | **Activity** | **Additional Comments** |
| --- | --- | --- |
|  |  |  |
